# Fractional Contribution of Wildland Firefighters’ Personal Protective Equipment on Physiological Strain

**DOI:** 10.3389/fphys.2018.01139

**Published:** 2018-08-14

**Authors:** Belén Carballo-Leyenda, José G. Villa, Jorge López-Satué, Pilar S. Collado, Jose A. Rodríguez-Marroyo

**Affiliations:** ^1^Department of Physical Education and Sports, University of León, León, Spain; ^2^Institute of Biomedicine, University of León, León, Spain; ^3^Empresa de Transformación Agraria, Madrid, Spain

**Keywords:** heat stress, thermal strain, thermophysiological response, core temperature, protective clothing

## Abstract

Activities performed by wildland firefighters are carried out wearing a personal protective equipment (PPE). Although the PPE protects workers from a wide variety of hazards, it may increase their physiological response and limit their performance. The aim of this study was to analyze the effect of the protective clothing (PPC) and the rest of the PPE elements (i.e., helmet, neck shroud, gloves, goggles, and mid-calf leather boots) on the wildland firefighters’ thermophysiological response during a moderate-intense exercise. Six male wildland firefighters performed, in a counterbalanced order, a 120 min graded exercise test wearing three different clothing configurations: (i) a traditional short sports gear (SG), (ii) a PPC, and (iii) a complete firefighters’ PPE. Trials were conducted on separate days at the same time of the day (12:00–15:00 h) and under climate-controlled conditions (∼30°C and ∼30% relative humidity). Heart rate, respiratory gas exchange, gastrointestinal and skin temperature, blood lactate concentration were recorded throughout the tests. Additionally, parameters of heat balance were estimated. Exercise time was shorter (*p* < 0.001) wearing the PPE (62.4 ± 13.3 min) than with the PPC (115.5 ± 5.0 min) and SG (118.2 ± 20.7 min). The increment of gastrointestinal temperature with the PPE (1.8 ± 0.3°C) was greater (*p* < 0.05) than the observed in PPC (1.2 ± 0.6°C) and SG (1.0 ± 0.2°C). The use of PPC increased (*p* < 0.05) subjects’ metabolic demand and skin temperature versus SG during the last 20 min of the test. The sweat retention in the PPE (1,045.7 ± 214.7 g) and PPC (978.3 ± 330.6 g) was significantly higher than that obtained in the SG (510.0 ± 210.0 g). Sweat efficiency decreased (*p* < 0.05) in the following order: PPE (45.6 ± 18.3%), PPC (64.3 ± 7.8%), and SG (79.3 ± 7.0%). These results highlight the importance of the PPE elements in the subjects’ thermal strain. The reduction in the sweat evaporation produced by the PPE, together with the ensemble mass caused a substantial increase in the subjects’ thermophysiological response. As a consequence the performance was reduced by ∼50%.

## Introduction

Wildfire firefighting is a demanding occupation (Rodríguez-Marroyo et al., 2012), which may require an average work energy expenditure of 2,400–3,000 kcal day^-1^, reaching even 12 kcal min^-1^ in specific moments (Cuddy et al., 2015). In addition, wildland firefighters may perform their work in adverse conditions that involve smoke and particulate matter inhalation (Adetona et al., 2016) and be exposed to both solar and flame radiation (Keiser and Rossi, 2008; Rodríguez-Marroyo et al., 2012), which increase their heat stress (González-Alonso et al., 1999; Cheuvront et al., 2010). Under these circumstances, the use of personal protective clothing (PPC) may exacerbate the wildland firefighters’ thermal strain (Carballo-Leyenda et al., 2017) and limit their performance. In general, the PPC protective characteristics may interfere with thermoregulation, reducing or preventing the heat transfer and sweat evaporation (Holmér, 2006; Cheung et al., 2010; Carballo-Leyenda et al., 2017). Therefore, the study of the wildland firefighters’ thermophysiological response to different PPC has acquired special relevance in recent years (Den Hartog et al., 2016; Carballo-Leyenda et al., 2017).

In some occupational settings the PPC is complemented by other elements, which complete the personal protective equipment (PPE). The PPE aims to protect workers from a wide variety of hazards and may be composed of various items such as helmets, boots, gloves and even a self-contained breathing apparatus, as in the case of structural firefighters (Selkirk and McLellan, 2004). The PPE may increase considerably, depending on its characteristics, the subjects’ physiological strain. Such is the case of structural firefighters’ PPE due to its high thermal insulation and mass, that can reach up to 26 kg (Barr et al., 2010). Therefore, the PPE metabolic and thermal impact in these workers has been widely studied in the literature (Sköldström, 1987; Smith et al., 1995; Selkirk and McLellan, 2004; Dreger et al., 2006; Bruce-Low et al., 2007; Taylor et al., 2012; Lee et al., 2014). However, there is a lack of knowledge about the fractional contribution that the different PPE elements have on the physiological strain of these subjects (Taylor et al., 2012; Lee et al., 2014). In this sense, the importance of boots in the increase of structural firefighters’ metabolic and thermal stress has been recently reported (Taylor et al., 2012; Lee et al., 2014). It has been observed this item may have a greater impact than that observed wearing the PPC (Taylor et al., 2012; Lee et al., 2014).

To our knowledge, there is a paucity of studies on the wildland firefighters’ PPE physiological impact (Budd et al., 1997; Den Hartog et al., 2016). Initial work of Budd et al. (1997) compared the thermophysiological response of two PPE with different thermal insulation levels. More recently, Den Hartog et al. (2016) analyzed the influence of several PPE on thermal balance according to different fabric composition and structure (i.e., wave and layers). The thermophysiological and subjective response of different PPC has also been recently studied (Carballo-Leyenda et al., 2017). Although the PPC studied in this research supposed a different pattern on the thermal balance parameters, the physiological response was not substantially modified (Carballo-Leyenda et al., 2017). This was probably due to the protocol performed. This study (Carballo-Leyenda et al., 2017) employed a protocol with a moderate metabolic rate, similar to those previously mentioned (Budd et al., 1997; Den Hartog et al., 2016). However, the need to increase the exercise intensity to accentuate the differences between PPC has been reported (Kofler et al., 2015; Fontana et al., 2017). Likewise, during wildfire suppression wildland firefighters may perform efforts at high intensities (Rodríguez-Marroyo et al., 2012; Cuddy et al., 2015). Consequently, knowing the PPE elements contribution on the wildland firefighters’ thermophysiological response at higher metabolic rates might help to set more effective passive heat dissipation strategies, such as work-rest cycles. Therefore, the aim of this study was to analyze the effect of the PPC and the rest of the PPE elements (i.e., helmet, neck shroud, gloves, goggles, and mid-calf leather boots) on the wildland firefighters’ thermophysiological and subjective response during a moderate-intense exercise.

## Materials and Methods

### Participants

Six trained and acclimated male wildland firefighters (mean ± SD; age: 30.6 ± 7.9 years, height: 1.77 ± 0.04 m, body mass: 75.1 ± 11.3 kg, maximal oxygen uptake: 53.7 ± 10.4 ml⋅kg^-1^⋅min^-1^, and body surface area: 1.8 ± 0.3 m^2^) participated in this study. All of them had more than 6 years of experience as elite wildland firefighters. During the last 5 months before the start the study, subjects trained 3–5 times per week (45–90 min/training session) as part of their scheduled training. Their training program was oriented to improve the muscular strength (1–2 sessions per week) and endurance (2–3 sessions per week). Many of these training sessions included work-specific activities (e.g., hiking, building fire lines, brush removal, and mopping up) where wildland firefighters wore the PPE and used specific tools (e.g., rakes, axes, swatters, shovels, and backpack pumps). This study was carried out in accordance with the recommendations of the Helsinki Conference for research on human subjects. The protocol was approved by the Ethics Committee of the University of León, Spain. All subjects gave written informed consent, prior to their participation in the study, in accordance with the Declaration of Helsinki.

### Experimental Design

Each subject performed four trials during four separate testing sessions. Trials were separated by at least 48 h, during which participants were asked to refrain from strenuous exercise, excessive sun exposure, and alcohol consumption. The first trial was a maximal incremental test to determine subjects’ maximal aerobic capacity (Bruce, 1971). During the second to fourth trial, subjects performed, in a counterbalanced design, a 120 min graded exercise test wearing three different clothing configurations: (i) a traditional short sports gear (SG) (i.e., shorts, cotton t-shirt, underwear, and socks), (ii) a PPC currently used by Spanish wildland firefighters (65% fire retardant viscose, 30% nomex and 5% kevlar, 1.5 kg, surface mass 270 g⋅m^-2^, thermal resistance 0.019 m^2^ K⋅W^-1^ and evaporative resistance 3.79 m^2^⋅Pa⋅W^-1^), and (iii) the complete Spanish wildland firefighters’ PPE (∼6 kg). This PPE includes the PPC and different items such as helmet, neck shroud, gloves, goggles, and mid-calf leather boots. The same clothing (i.e., cotton t-shirt, briefs, and socks) was worn under PPE and PPC. In addition, the same running shoes (250–300 g per shoe) were used with SG and PPC. During all tests, to simulate a real scenario subjects wore a backpack pump (20 kg), which is routinely used during wildfire firefighting (Rodríguez-Marroyo et al., 2012). The total ensemble mass was 21.0 ± 0.1, 22.7 ± 0.2, and 26.8 ± 0.6 kg for SG, PPC, and PPE, respectively.

### Tests Protocol

All tests were performed on a treadmill (h/p/cosmos pulsar, h/p/cosmos sports and medical GMBH, Nußdorf-Traunstein, Germany). Each test was preceded by a 10 min warm-up at 60% of maximum heart rate (HR) (8–10 km⋅h^-1^) and 5 min of stretching. In the first testing session, subjects performed a maximal test according to the protocol described by Bruce (1971). The test started with a speed of 2.5 km⋅h^-1^ and a slope of 10%. The speed and grade were incremented every 3 min until volitional exhaustion.

The 120 min graded exercise tests were performed at the same time of the day (12:00–15:00 h) in a laboratory under climate-controlled conditions (room temperature ∼30°C, relative humidity ∼30%, air pressure ∼692 mm Hg), simulating those analyzed in real wildfires (Rodríguez-Marroyo et al., 2012). The experimental protocol consisted of six sets of walking at 6 km⋅h^-1^ with a gradual increase of the slope (1, 2, 5, 8, 10, and 13%) and 5 min passive recovery periods in between. Each set duration was 15 min, except for the first one that was 20 min. During recovery periods, 0.15 ml⋅kg^-1^ of water every 1 min of exercise at 15°C (Selkirk and McLellan, 2004) was administered to prevent an effect of dehydration on sweat rate (Cheuvront et al., 2010). The protocol used in this study was based on previous studies (Selkirk and McLellan, 2004; Carballo-Leyenda et al., 2017) and the selected speed and slope allowed subjects to perform an exercise intensity >70% of the maximal HR, which simulates wildland firefighters’ moderate to high working conditions achieved during wildfire suppression (Rodríguez-Marroyo et al., 2012).

### Measurements

ECG monitoring (Medisoft MedCard, Medisoft Group, Sorinnes, Belgium) was performed throughout Bruce’s test to detect heart problems. During all trials, the HR response and the respiratory gas exchange was continuously measured every 5 s (RS800, Polar Electro Oy, Kempele, Finland) and breath-by-breath (Medisoft Ergocard, Medisoft Group, Sorinnes, Belgium), respectively. VO_2max_ was accepted as the highest 30-s moving average.

Gastrointestinal temperature (T_gi_) was recorded throughout experimental trials using a Jonah intestinal temperature capsule (VitalSense, Phillips Respironics, Bend, OR, United States), which was ingested at least 8 h before the beginning of trials (Larsen et al., 2015). Skin temperature (T_skin_) was measured using dermal patches (VitalSense, Phillips Respironics, Bend, OR, United States) placed in three sites: in the chest at the height of the left major pectoral, in the right anterior hip and in the right anterior thigh. Mean T_skin_ was calculated using a modified version of Burton (1935) using standard skin surface area weighting coefficients as described by Hardy and DuBois (1938):

1 $${\text{T}}_{\text{skin}}=0.60\times {\text{T}}_{\text{chest}}+0.20\times {\text{T}}_{\text{hip}}+0.20\times {\text{T}}_{\text{thigh}}$$

T_gi_, T_skin_, as well as HR and VO_2_ data from the last 5 min of each exercise stage, were considered representative measurements of the entire stage. The T_gi_ and HR were used to calculate the physiological strain index (PSI) throughout the trials according to Tikuisis et al. (2002). Capillary blood samples were taken from the earlobe to measure blood lactate concentration (Lactate Scout, Senslab, Leipzig, Germany) after the end of each exercise set.

During the last 30-s of each exercise stage, the rating of perceived exertion (RPE) was recorded using the Borg scale (6–20) (Borg, 1982). The scale was explained and administered by the same researcher, asking about subjects’ perceived exertion using the same question. A cue card was located in front of subjects to allow immediate reference to the scale. Additionally, subjects’ thermal sensation was recorded at the end of each exercise bout, using a categorical scale (2–8) (Havenith and Heus, 2004). Verbal anchors associated with 2 and 8 were identified with *comfortably warm* and *very hot*, respectively.

Subjects, in underwear, and each clothing component were separately weighted (50K150, COBOS, Hospitalet de Llobregat, Barcelona, Spain) at the beginning and the end of each trial. This allows calculating the total sweat production, sweat residue, and sweat evaporation (Havenith and Heus, 2004; Kofler et al., 2015). Total sweat was corrected for the fluid intake. Finally, the sweat efficiency was calculated as the ratio between sweat evaporation and total sweat (Havenith and Heus, 2004).

Heat balance of the body was estimated using a method of partitional calorimetry summarized in Eq. (2) (Bröde et al., 2008). This estimation was included to support the physiological variables analyzed, despite its limitation. It has been shown that the two-compartmental thermometry model may systematically underestimate the body heat storage (Jay et al., 2007) due to: (i) the thermal influences of the muscle tissue are not considered independently of the “core” and “shell” (Jay et al., 2007) and (ii) the assumption that a common specific heat capacity is applied to all individuals, irrespective of body composition (Taylor et al., 2014).

2 $$\text{S}=\text{M}-\text{W}\pm \text{DRY}-{\text{E}}_{\text{sk}}-\text{RES}$$

Components of the equation were heat storage (S), metabolic energy production (M), effective mechanical work (W), heat loss through evaporative and convective heat exchange via respiration (RES = E_res_ + C_res_), evaporative heat loss (E_sk_), and dry heat loss (DRY = C + R + K). All heat balance parameters were calculated in W⋅m^-2^. The components were estimated and served only to substantiate the results. The rate of metabolic heat production was calculated from measured respiratory quotient (RQ) and VO_2_ (L⋅min^-1^) and the body surface area (A_D_; m^2^) calculated using DuBois formula (DuBois and DuBois, 1916), as shown below in Eq. (3) (Gagge and Gonzalez, 1996):

3 $$\text{M}=[0.23(\text{RQ})+0.77]\times 5.873\times {\text{VO}}_{2}\times (60/{\text{A}}_{\text{D}})$$

Effective mechanical work was calculated using acceleration due to gravity (9.8 m⋅s^-2^), the dressed mass of participants (m; kg), the speed (v; m⋅s^-1^) and the grade fraction (F) of the treadmill and A_D_, using Eq. (4) (Givoni and Goldman, 1972):

4 $$\text{W}=9.8\times \text{m}\times \text{v}\times \text{F}\times {\text{A}}_{\text{D}}{}^{-1}$$

The respiratory heat loss components C_res_ and E_res_ were calculated using Eqs (5) and (6), respectively (Bröde et al., 2008):

5 $${\text{C}}_{\text{res}}=1.516\times 10^{-3}\times \text{M}\times (28.56-0.641\times {\text{P}}_{\text{a}}-0.885\times {\text{T}}_{\text{a}})$$

6 $${\text{E}}_{\text{res}}=1.27\times 10^{-3}\times \text{M}\times (59.34-11.63\times {\text{P}}_{\text{a}}-0.53\times {\text{T}}_{\text{a}})$$

where P_a_ is the atmospheric pressure in Pascals, T_a_ is the ambient temperature in °C and M is the rate of metabolic heat production in W⋅m^-2^, calculated with Eq. (3).

S was calculated as (ΔT_b_ × Δt^-1^) × BM × A_D_^-1^ × c_p_. The rate of change of body temperature (T_b_) for test duration (s) was ΔT_b_ × Δt^-1^ in °C⋅s^-1^, where c_p_ represented the specific heat of body tissue (3,480 J⋅kg^-1^⋅°C^-1^) and BM, body mass in kg. Mean body temperature (T_b_) in °C was estimated by 4:1 ratio of gastrointestinal temperature (T_gi_) and T_skin_ as T_b_ = 0.8 × T_gi_ + 0.2 × T_skin_, recommended for warm environments (Bröde et al., 2008).

E_sk_ corrected for the respiratory loss was estimated as λ × (m_e_ × Δt^-1^) × A_D_^-1^ - E_res_. Where m_e_ is the evaporative sweat loss (g) with Δt denoting measurement time (s), λ the enthalpy of evaporation (2,430 J⋅g^-1^) and E_res_ is the respiratory heat evaporation calculated using Eq. 6. DRY resulted solving the heat balance equation with the other known components using Eq. (7) (Bröde et al., 2008):

7 $$\text{DRY}=\text{M}-\text{W}-{\text{E}}_{\text{sk}}-\text{RES}-\text{S}$$

Additionally, total thermal insulation of clothing (It) was estimated through the equation (T_skin_ - T_a_) × DRY^-1^ (Bröde et al., 2008). Finally, the heat strain index (HSI) was calculated as an estimate of the thermal compensability of the environment. An HSI > 1.0 indicated uncompensable heat stress, and an HSI < 1.0 indicated compensable heat stress (McLellan et al., 2013). The HSI was calculated as the ratio of the required evaporative cooling for heat balance (E_req_, in W⋅m^-2^) and the maximal evaporative capacity of the environment (E_max_, in W⋅m^-2^) (McLellan et al., 2013). E_req_ was calculated using the thermal balance equation parameters in the following manner (McLellan et al., 2013):

8 $${\text{E}}_{\text{req}}=\text{M}-\text{W}\pm \text{DRY}\pm \text{RES}$$

E_max_ was calculated according to the equation of McLellan et al. (2013):

9 $${\text{E}}_{\max}=16.5\times {\text{i}}_{\text{m}}^{}\times {\text{I}}_{\text{t}}{}^{-1}\times ({\text{P}}_{\text{sk}}-{\text{φ}}_{\text{a}}\times {\text{P}}_{\text{a}})$$

where 16.5 is the Lewis number (°C⋅kPa^-1^), i_m_ is the Woodcock water vapor permeability coefficient (dimensionless) estimated with a heated and wetted articulated manikin, P_sk_ and P_a_ are the skin saturation vapor pressure and the ambient saturation vapor pressure in Kilopascals, and φ_a_ is the ambient relative humidity.

### Statistical Analysis

The results are expressed as mean ± standard deviation (SD). The assumption of normality was verified using the Shapiro–Wilk’s test. The variables analyzed throughout the trials (VO_2_, ventilation, HR, blood lactate concentration, T_gi_, T_skin_, PSI, RPE, and TS) were compared using a repeated two-way ANOVA with two within-subject factors (clothing and time). A one-way ANOVA with repeated measures was applied to calculate differences between different parameters of heat balance, HSI, and sweat. When a significant *F*-value was found, Bonferroni’s test was used to establish significant differences between means. The assumption of sphericity was checked using the Mauchly’s test, when this assumption was violated the Greenhouse–Geisser adjustment was performed. Partial eta-squared (${\text{η}}_{\text{p}}^{2}$) was calculated for the dependent variables as a measure of effect size. Values of 0.01, 0.06, and 0.14 were considered small, moderate, and large, respectively (Lakens, 2013). The relationship between variables was determined using the Pearson correlation coefficient (*r*). Values of *p* < 0.05 were considered statistically significant. SPSS V.19.0 statistical software (SPSS, Inc., Chicago, IL, United States) was used.

## Results

**FIGURE 1:**
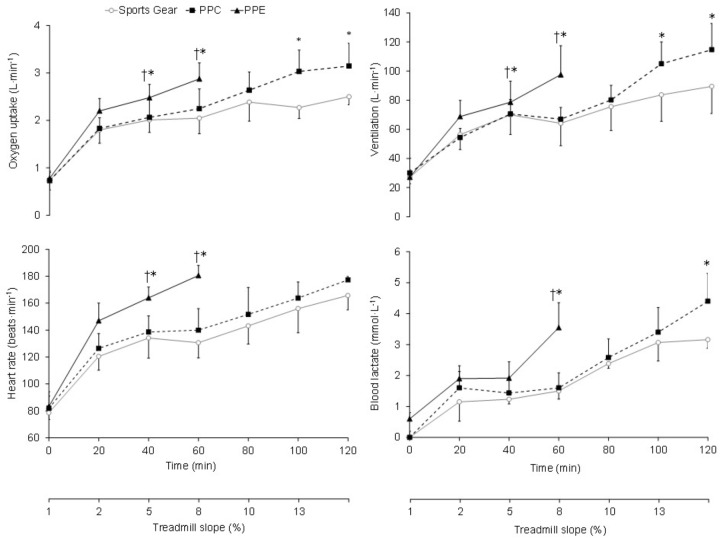
Comparative responses of oxygen uptake, ventilation, heart rate (HR), and blood lactate concentration wearing the short sports gear, the personal protective clothing (PPC) and the personal protective equipment (PPE) during the 120 min graded test. Values are mean ± SD. ^†^Differences with PPC (*p* < 0.05). ^∗^Differences with sports gear (*p* < 0.05).

The trial duration was significantly shorter (*p* < 0.001) when subjects wore the PPE [62.4 ± 13.3 min (range: 35–72 min)] than when they wore the PPC [115.5 ± 5.0 min (range: 90–120 min), ${\text{η}}_{\text{p}}^{2}$ = 0.96] and the SG [118.2 ± 20.7 min (range: 110–120 min), ${\text{η}}_{\text{p}}^{2}$ = 0.77]. There was a significant interaction between clothing condition and time for ergospirometry variables (**Figure 1**). The highest (*p* < 0.05) VO_2_, ventilation and HR at minute 40 (${\text{η}}_{\text{p}}^{2}$= 0.49–0.90) and 60 (${\text{η}}_{\text{p}}^{2}$= 0.45–0.93) were analyzed with the PPE. The PPC condition significantly increased (*p* < 0.05) the VO_2_ and ventilation versus the SG in the last 20 min of the test (${\text{η}}_{\text{p}}^{2}$= 0.29–0.65). The highest (*p* < 0.05) blood lactate concentration at 60 and 120 min was found wearing the PPE (${\text{η}}_{\text{p}}^{2}$ = 0.51 and 0.57) and PPC (${\text{η}}_{\text{p}}^{2}$= 0.58), respectively (**Figure 1**).

**FIGURE 2:**
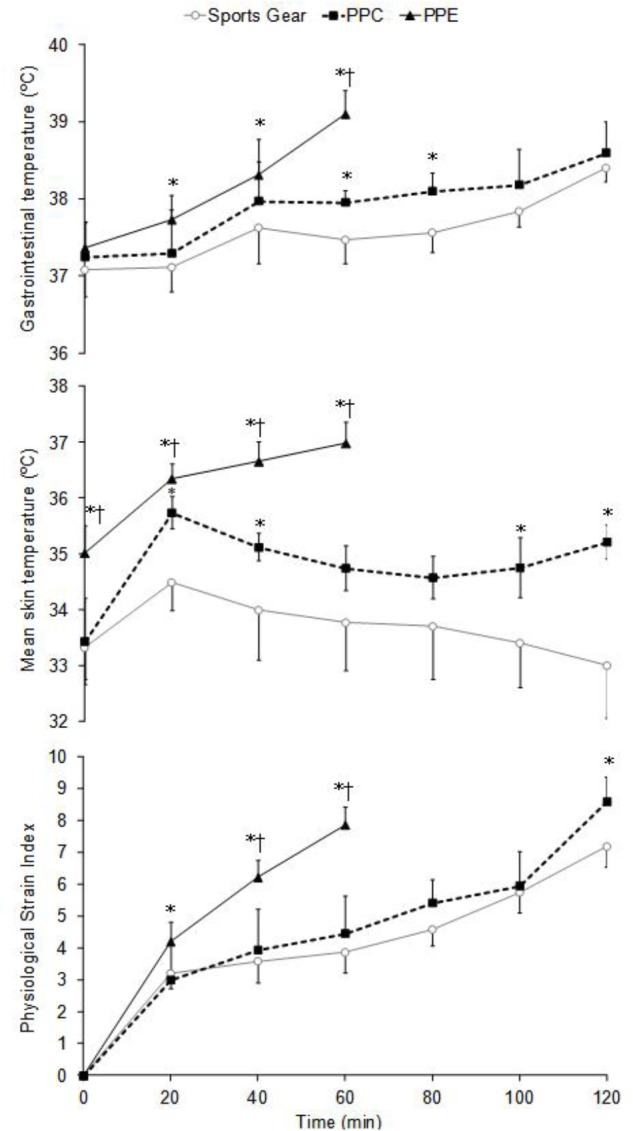
Gastrointestinal and skin temperature and physiological strain index pattern recorded during the trials wearing the short sports gear, the PPC and the PPE. Values are mean ± SD. ^†^Differences with PPC (*p* < 0.05). ^∗^Differences with sports gear (*p* < 0.05).

Wearing the PPE resulted in a higher T_gi_ (*p* < 0.05, ${\text{η}}_{\text{p}}^{2}$= 0.31–0.77), T_skin_ (*p* < 0.001, ${\text{η}}_{\text{p}}^{2}$ = 0.74–0.87), and PSI (*p* < 0.05; ${\text{η}}_{\text{p}}^{2}$ = 0.16–0.87) throughout the test versus those analyzed with the SG. Similarly, T_skin_ was significantly higher (*p* < 0.05, ${\text{η}}_{\text{p}}^{2}$= 0.31–0.86) with the PPE than the PPC from the beginning of the test (**Figure 2**). The pattern of this variable changed markedly between PPC and SG during the last 20 min of the test (*p* < 0.05; ${\text{η}}_{\text{p}}^{2}$ = 0.46 and 0.88). The trial duration was significantly (*p* < 0.001) correlated with the T_gi_ (*r* = -0.76) and T_skin_ (*r* = -0.80) increase rate. There were significant differences wearing the PPE and PPC for the PSI at minute 40 (*p* < 0.05, ${\text{η}}_{\text{p}}^{2}$ = 0.54) and 60 (*p* < 0.001, ${\text{η}}_{\text{p}}^{2}$ = 0.75). Likewise, the PSI was greater with the PPC than the SG at the end of the test (*p* < 0.05; ${\text{η}}_{\text{p}}^{2}$ = 0.38) (**Figure 2**).

**FIGURE 3:**
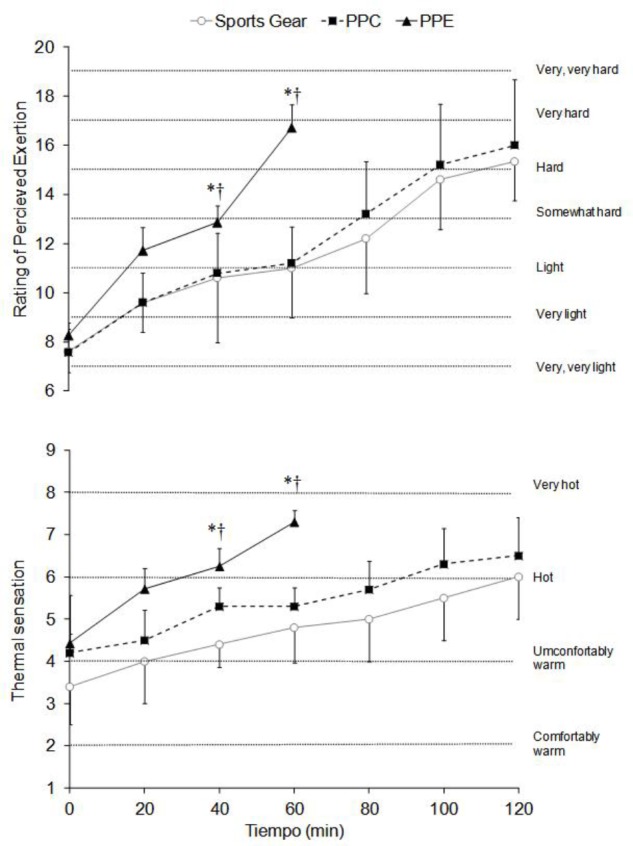
Rating of perceives exertion and thermal sensation wearing the short sports gear, the PPC and the PPE. Values are mean ± SD. ^†^Differences with PPC (*p* < 0.05). ^∗^Differences with sports gear (*p* < 0.05).

From the 40^th^ minute there was a significant increase (*p* < 0.001) in the RPE (${\text{η}}_{\text{p}}^{2}$ = 0.66–0.82) and thermal sensation (${\text{η}}_{\text{p}}^{2}$ = 0.65–0.81) wearing the PPE (**Figure 3**). Finally, both the perceptual (i.e., RPE and thermal sensation) and physiological variables (i.e., VO_2_, ventilation, HR, blood lactate, T_gi_, T_skin_, and PSI), except the T_skin_ in SG condition, increased significantly (*p* < 0.05, ${\text{η}}_{\text{p}}^{2}$ = 0.55–0.98) throughout the trials (**Figures 1**–**3**).

Although the highest sweat rate was found with the PPE (*p* < 0.05, ${\text{η}}_{\text{p}}^{2}$ = 0.22 and 0.66), the total sweat production was similar for all clothing conditions (**Table 1**). The sweat retained in the PPE and PPC was significantly higher than that obtained in the SG (*p* < 0.05, ${\text{η}}_{\text{p}}^{2}$ = 0.60 and 0.42, respectively). The lowest (*p* < 0.05) sweat evaporation and sweat efficiency were obtained wearing the PPE (${\text{η}}_{\text{p}}^{2}$ = 0.34–0.61). In addition, significant differences between PPC and SG for sweat efficiency was found (*p* < 0.05; ${\text{η}}_{\text{p}}^{2}$ = 0.51).

**Table 1:** Sweat measurements analyzed in the study (mean ± SD).

	PPE	PPC	Sports gear
Total sweat production (g)	2,045.1 ± 553.3	2,720.0 ± 566.5	2,426.7 ± 360.8
Sweat rate (g⋅h^-1^)	1,924.6 ± 291.2^∗†^	1,421.0 ± 401.9	1,213.3 ± 180.4
Sweat residue (g)	1,045.7 ± 214.7^∗^	978.3 ± 330.6^∗^	510.0 ± 210.0
Sweat evaporation (g)	999.4 ± 493.4^∗†^	1,741 ± 395.5	1,916 ± 286.4
Sweat efficiency (%)	45.6 ± 18.3^∗†^	64.3 ± 7.8^∗^	79.3 ± 7.0

*PPE, personal protective equipment. PPC, personal protective clothing. ^†^Differences with PPC (p < 0.05). ^∗^Differences with sports gear (p < 0.05).*

Estimated parameters of heat balance are shown in **Table 2**. As expected, wearing the full protective equipment increased thermal insulation compared to PPC (*p* < 0.001, ${\text{η}}_{\text{p}}^{2}$ = 0.55). In addition, the high metabolic heat production and the low dry heat exchange observed with this clothing configuration resulted in the highest heat storage (*p* < 0.001, ${\text{η}}_{\text{p}}^{2}$ = 0.82) and involved the highest HSI (*p* < 0.001, ${\text{η}}_{\text{p}}^{2}$ = 0.76).

**Table 2:** Estimated parameters of heat balance analysis (mean ± SD).

	PPE	PPC	Sports gear
Heat storage (W⋅m^-2^)	67.8 ± 13.8^∗†^	31.8 ± 13.1	19.0 ± 9.1
Net metabolic heat production (W⋅m^-2^)	374.9 ± 44.3^∗^	307.5 ± 57.9	293.0 ± 38.9
Respiratory heat exchange (W⋅m^-2^)	17.6 ± 2.5^∗†^	14.3 ± 2.3	13.7 ± 1.4
Evaporative heat loss from skin (W⋅m^-2^)	321.8 ± 59.6	295.8 ± 88.2	324.7 ± 29.7
Dry heat exchange (W⋅m^-2^)	27.0 ± 2.9^∗^	34.1 ± 8.3^∗^	74.5 ± 7.4
Total clothing insulation (m^2^⋅°C⋅W^-1^)	0.240 ± 0.034^∗†^	0.153 ± 0.050^∗^	0.055 ± 0.018
Heat stress index	2.6 ± 0.4^∗†^	1.4 ± 0.3^∗^	0.5 ± 0.2

*PPE, personal protective equipment; PPC, personal protective clothing. ^†^Differences with PPC (p < 0.05). ^∗^Differences with sports gear (p < 0.05).*

## Discussion

The main finding of this study was that the use of PPE in moderate-high efforts leads to a significant increase in the wildland firefighters’ physiological strain. This physiological strain was due mainly to the impact that the helmet, neck shield, gloves, and boots had in sweat evaporation and thermal insulation. This fact caused a significant reduction in the subjects’ performance compared to that analyzed when the PPC and the SG were worn.

The performance reduction caused by the PPE compared to the other conditions was ∼50%. This reduction in the time of effort was similar to that previously reported in structural firefighters (Montain et al., 1994; McLellan et al., 1996; Taylor et al., 2012), where the PPC’s thermal insulation (∼0.47 m^2^⋅K⋅W^-1^) (Holmér et al., 2006) was substantially higher than the one of the present study (∼0.23 m^2^⋅K⋅W^-1^) (Raimundo and Figueiredo, 2009). Collectively, our results seem to indicate the high impact that PPE may have on the wildland firefighters’ thermophysiological strain. In fact, wearing the PPC only meant a decrease in subjects’ performance of 17% compared to the use of the SG. This result was higher than that obtained (10%) by Fogarty et al. (2004) when the structural firefighters’ PPC was studied in warm conditions (40°C). Kofler et al. (2015) also obtained a performance reduction of 10% with the use of a thermal protection suit, very similar to the one analyzed in this study, in temperate conditions (25°C). Possibly the high metabolic rate reached by our subjects (443.6 ± 41.4, 351.6 ± 57.3, and 336.7 ± 35.1 W⋅m^-2^ wearing the PPE, PPC, and SG, respectively) determined the obtained results. There were no substantial differences in the VO_2_ response with the use of PPC and SG at moderate metabolic rates (**Figure 1**). This agrees with recent results where it has been shown that wearing PPC did not cause a significant increase in subjects’ physiological response during a moderate exercise intensity (Carballo-Leyenda et al., 2017).

Wearing the PPE resulted in an average rise in VO_2_ of ∼20% compared to SG condition, and it was significantly more pronounced at the end of the test (∼30 and ∼45% at 40 and 60 min, respectively) (**Figure 1**). This might be related to the higher PPE weight (∼ 6 kg) (Dorman and Havenith, 2009; Lee et al., 2014). This would also explain the greater increases observed (>20%) with the PPE in structural firefighters, where the use of self-contained breathing apparatus may increase the ensemble weight up to 26 kg (Sköldström, 1987; Smith et al., 1995; Dreger et al., 2006; Taylor et al., 2012). On the other hand, the boots might contribute significantly to the increase in the PPE metabolic cost. The importance of the weight distribution with respect to the center of gravity in the subjects’ physiological response has been reported (Dorman and Havenith, 2009; Taylor et al., 2012; Lee et al., 2014). As a result, it has been shown that structural firefighters’ boots (∼2.5 kg) may suppose an increase in metabolic cost up to ∼11% (Taylor et al., 2012). Therefore, it may be thought that the boots used in this study (∼2.0 kg) might have contributed significantly to increase the VO_2_. Finally, the subjects in this study completed the PPE test reaching ∼75% of VO_2max_. This circumstance has been previously observed in structural firefighters (Sköldström, 1987; Smith et al., 1995; Taylor et al., 2012; Lee et al., 2014) and it may be related to the muscle fatigue (Lucía et al., 1999) achieved with this configuration. However, it would also be plausible to think that the PPE thermal insulation involved an excessive increase in body temperature (**Figure 2**), which the subjects might not compensate and as a result they had to cease the effort to not compromise their health (Nielsen et al., 1993; González-Alonso et al., 1999).

The highest HR was obtained in PPE condition (**Figure 1**). HR was on average ∼20 bpm higher with this configuration, reaching ∼80% of the maximal HR already during the first exercise stage. This increase in the HR response might be a consequence of the metabolic and thermoregulatory demand imposed by the PPE (Ely et al., 2010; Cuddy et al., 2014), as well as the direct effect that temperature has on HR (Jose et al., 1970). Wearing the PPE is associated with a thermoregulatory restriction that implies an increase in the cutaneous blood flow to enhance the body heat elimination, increasing the transfer of dry heat and sweating (Smith et al., 1995; Selkirk and McLellan, 2004; Barr et al., 2010). The HR pattern during the trials performed with the PPC and SG was similar. This highlights the influence that the boots, gloves, helmet, and neck covers had on the physiological strain analyzed in the present study. Do not use these elements increase the body surface exposed to the environment and facilitate the heat loss (Holmér, 2006; Lee et al., 2014). In addition, the low thermal insulation of the PPC used in this study allowed a greater heat dissipation (Carballo-Leyenda et al., 2017).

The increase in T_gi_ found in this study (**Figure 2**) seems to indicate that the subjects reached a situation of uncompensable heat stress during the tests (Den Hartog et al., 2016). It has been previously reported that a temperature above 38°C may be a limiting factor of performance during exercise in the heat (González-Alonso et al., 1999). All T_gi_ analyzed at the end of the tests exceeded this value (∼39 and ∼38.5°C with the PPE and PPC and SG, respectively). However, the increase in core temperature was approximately twice as fast in PPE condition (0.028 ± 0.007°C min^-1^) compared to PPC (0.012 ± 0.004°C min^-1^) or SG (0.011 ± 0.004°C min^-1^). This occurred as a result of the combined effect of the increased metabolic heat production and the heat dissipation limitation associated with the use of PPE, which resulted in a marked situation of uncompensable heat stress (**Table 2**) and provoked a significantly increased both T_gi_ and T_skin_ (**Figure 2**) (Sköldström, 1987; Montain et al., 1994; Smith et al., 1995; Bruce-Low et al., 2007; Wen et al., 2015; Fontana et al., 2017). This fact contributed substantially to reduce the test time to almost half when the subjects wore the PPE. We found relationships of *r* = -0.76 and -0.80 between the exercise duration and the T_gi_ and T_skin_ increase, respectively.

The observed T_skin_ (**Figure 2**) emphasize the loss of heat dissipation efficiency due mainly to the use of PPE. The average temperature increase with this configuration was 1.89 ± 0.48°C, which was twofold greater than the value observed with PPC (0.77 ± 0.34°C). On the contrary, this data was lower than that described in other studies (∼3°C) with structural firefighters (Sköldström, 1987; Smith et al., 1995; Bruce-Low et al., 2007; Fontana et al., 2017) or wearing chemical protective suits (Wen et al., 2015). A T_skin_ above 35°C and a reduced T_gi_-T_skin_ gradient have been associated with a significant increase in peripheral blood flow, which might limit the subjects’ aerobic performance, even in the absence of dehydration (Ely et al., 2010; Cuddy et al., 2014; Faulkner et al., 2015). T_skin_ values above 35°C were observed wearing the PPE since the beginning of the test (**Figure 2**), in addition, the T_gi_-T_skin_ gradient in this configuration was lower (1.30 ± 0.30°C) than the one analyzed with PPC (3.02 ± 0.58°C) and SG (4.13 ± 0.44°C). All this might have contributed to increase substantially the cardiovascular strain during the PPE configuration and might justify the slight increase in HR when the subjects wore the PPC (Cuddy et al., 2014).

As a consequence of the cardiovascular and thermal pattern analyzed in this study, the RPE, thermal sensation and PSI obtained when wearing PPE were significantly higher than wearing the PPC and SG. The RPE pattern found (**Figure 3**) throughout the trials might be related to the cardiovascular load analyzed (Sköldström, 1987; Bruce-Low et al., 2007). However, the body heat storage caused by the increase of both T_gi_ and T_skin_ (**Figure 2**) might affect the thermal sensation values obtained (Sköldström, 1987; Smith et al., 1995; Bruce-Low et al., 2007; Kofler et al., 2015). The wildland firefighters’ physiological strain according to the mean PSI (∼6.0) was moderate. This value was higher than the one reported previously in real wildfires (∼4.5) (Rodríguez-Marroyo et al., 2012) and similar to that reported in structural firefighters wearing the PPE (∼6.0) during short duration and moderate intensity trials (Petruzzello et al., 2009). The PSI at the end of the tests in the different experimental conditions was similar (∼8.0), and it was cataloged as *high*. However, wearing the PPE (**Figure 2**) supposed that subjects achieved this values in the half of time. In this way, when the maximum PSI was obtained with the PPE, approximately a half of the value was analyzed with the PPC or SG (∼3.5).

As suggested above, the impact of PPE on thermal insulation and sweat evaporation determined the wildland firefighters’ thermophysiological and subjective response (Sköldström, 1987; Holmér, 2006; Caldwell et al., 2011; Lee et al., 2014; Wen et al., 2015). While wearing the PPC (i.e., 88% of the body surface) led to a reduction in evaporative efficiency of 19%, adding the other PPE elements (i.e., helmet, neck shroud, gloves, boots, 12% of the surface body) caused an additional loss of efficiency of 28% (**Table 1**). This circumstance highlights the importance of these elements in the sweat evaporation. In this sense, the sweat evaporated during the PPE test was 43% lower than the one found with the PPC, while no difference between PPC and SG was observed. These results are in agreement with those previously obtained in military settings (Montain et al., 1994; Caldwell et al., 2011). Montain et al. (1994) found a decrease in evaporation of ∼50% when compared a fully clothed ensemble versus a partial clothing ensemble during an intense exercise under hot conditions. The same finding was obtained by Caldwell et al. (2011) when comparing a combat body armor with the helmet or with cloth hat in hot conditions.

The increase in thermal insulation between SG *vs.* PPC and PPC *vs.* PPE was similar (∼0.1 m^2^⋅°C⋅W^-1^) (**Table 2**). This fact confirms the relationship between the increase in thermal insulation and the covered body surface (Nunneley, 1989; Havenith, 1999; Holmér, 2006; McLellan et al., 2013). However, our results show the importance of the elements added to the PPC in the heat dissipation limitation, in spite of the small body surface that they possess. Mainly, this might be related to the use of the helmet, since the head is a zone of high thermoregulatory efficiency due to its high surface/volume ratio (Rasch et al., 1991; Lee et al., 2014), its dense vascularization, the lack of vasoconstriction of the head’s skin, and its minimal adiposity that makes for high thermoconductivity (Shvartz, 1970; Cheung, 2007). Under the conditions reproduced in our study, using the PPE led to a high impact on the thermal balance. The heat storage in the PPE was twofold the estimated with the PPC (**Table 2**). This fact may be of special relevance during wildfire suppression since wildland firefighters may spend up to ∼1 h in moderate-high intensity zones (i.e., >70% maximal HR) (Rodríguez-Marroyo et al., 2012). In this scenario, these professionals may be exposed to a high risk of hyperthermia and heat exhaustion, compromising their health and safety.

In summary, the thermal insulation produced by the PPE led to a reduction in the sweat evaporation, causing a substantial increase in the subjects’ thermophysiological response. This fact markedly reduced the wildland firefighters’ effort time (∼50%). Our results highlight the importance of the helmet, neck shroud, gloves and boots in the subjects’ thermal strain.

## Author Contributions

BC-L, JV, and JR-M: study design and interpretation of the results. BC-L, JV, JL-S, and PC: data collection. BC-L, JV, and PC: data analyses. BC-L and JR-M: manuscript writing. BC-L, JV, JL-S, PC, and JR-M: approved the final manuscript version.

## Conflict of Interest Statement

The authors declare that the research was conducted in the absence of any commercial or financial relationships that could be construed as a potential conflict of interest.
